# Zinc binding to RNA recognition motif of TDP-43 induces the formation of amyloid-like aggregates

**DOI:** 10.1038/s41598-017-07215-7

**Published:** 2017-07-28

**Authors:** Cyrille Garnier, François Devred, Deborah Byrne, Rémy Puppo, Andrei Yu. Roman, Soazig Malesinski, Andrey V. Golovin, Régine Lebrun, Natalia N. Ninkina, Philipp O. Tsvetkov

**Affiliations:** 1 Mécanismes Moléculaires dans les Démences Neurodégénératives, Université de Montpellier, EPHE, INSERM, U1198, F-34095 Montpellier, France; 2 Université de Rennes 1, Campus de Beaulieu, 35042 Rennes cedex, France; 3 Aix-Marseille Univ, Inserm, CRO2 UMR_S 911, Faculté de Pharmacie, 13385 Marseille, France; 4 Institut de Microbiologie de la Méditerranée, CNRS, FR3479, Aix-Marseille Université, Marseille, France; 5 Institute of Physiologically Active Compounds, RAS, 142432 Chernogolovka, Russian Federation; 6 Lomonosov Moscow State University, Moscow, 119991 Russian Federation; 7 School of Biosciences, Cardiff University, Sir Martin Evans Building, Museum Avenue, Cardiff, CF10 3AX, UK

## Abstract

Aggregation of TDP-43 (transactive response DNA binding protein 43 kDa) is a hallmark of certain forms of amyotrophic lateral sclerosis (ALS) and frontotemporal lobar degeneration (FTLD). Moreover, intracellular TDP-43-positive inclusions are often found in other neurodegenerative diseases. Recently it was shown that zinc ions can provoke the aggregation of endogenous TDP-43 in cells, allowing to assume a direct interaction of TDP-43 with zinc ions. In this work, we investigated zinc binding to the 102–269 TDP-43 fragment, which comprise the two RNA recognition motifs. Using isothermal titration calorimetry, mass spectrometry, and differential scanning fluorimetry, we showed that zinc binds to this TDP-43 domain with a dissociation constant in the micromolar range and modifies its tertiary structure leading to a decrease of its thermostability. Moreover, the study by dynamic light scattering and negative stain electron microscopy demonstrated that zinc ions induce auto-association process of this TDP-43 fragment into rope-like structures. These structures are thioflavin-T-positive allowing to hypothesize the direct implication of zinc ions in pathological aggregation of TDP-43.

## Introduction

TDP-43 is a highly conserved nuclear protein that regulates multiple processes in the cell nucleus and cytoplasm, and which could be involved in apoptosis and cell division^1^. In particular, it has been shown to play a role in all steps of mRNA life cycle and to regulate non-coding RNAs^2^. Mutations in TARDBP, the TDP-43 encoding gene, are linked to familial forms of amyotrophic lateral sclerosis (ALS)^3–5^. Aggregated TDP-43s often form cytoplasmic neuronal inclusions in both familial and sporadic forms of ALS as well as in the most common variant of frontotemporal lobar degeneration (FTLD)^6–8^. Moreover, there is a growing body of evidence implicating TDP-43 in Alzheimer’s disease (AD)^9^. Intracellular TDP-43 inclusions are present in up to 57% of AD cases and their presence has robust connection with AD clinical phenotype^10^. In addition, TDP-43 is also able to form cross-seeding amyloid oligomers with Alzheimer’s amyloid-β^11^. The molecular mechanism of TDP-43 aggregation is not completely elucidated, although several models have been proposed^12–16^. This aggregation is often associated with numerous post-translational modifications found in pathological TDP-43 from brains of ALS patients^17^ or mutations in C-terminal domain of TDP-43^18^. Such mutations not only accelerate TDP-43 aggregation but also promote its toxicity *in vivo* ^19^.

Recently, it has been demonstrated that zinc is able to induce aggregation of endogenous TDP-43 in cells^20^. Zinc has been linked to pathogenesis of a number of neurodegenerative diseases including AD^21^. Indeed, zinc ions bind aggregate-prone proteins such as amyloid-β, tau, FUS/TLS, and superoxide dismutase 1 (SOD1), and often promote their aggregation^22^. The molecular mechanism of zinc-induced aggregation of amyloid-β in AD was extensively studied over the past years^23–26^, which led to the development of effective inhibitors of amyloid-β aggregation^27^. Thus, it is important to understand whether zinc ions are also able to trigger TDP-43 aggregation by a direct mechanism, i.e. by interacting with this protein and favoring its aggregation.

**Figure 1:**
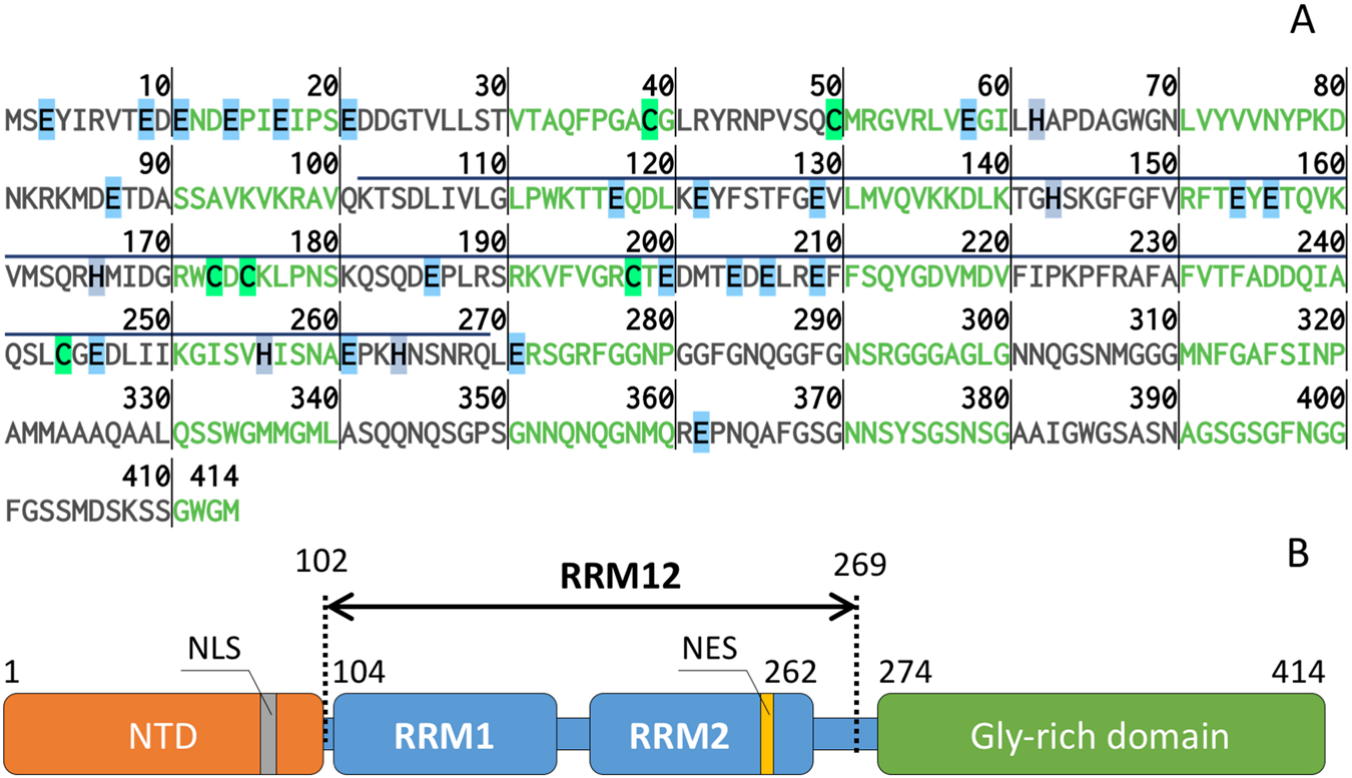
(**A**) Amino acid sequence of Transactive response DNA-binding protein 43 (TDP-43). The fragment of 102–269 (RRM12) used in this study is overlined. The amino acids, which are able to chelate zinc ions are highlighted: in light blue – Glu, in green – Cys and in grey – His. Image was generated using Protein Sequence Analysis Tool^54^. (**B**) Domain organization of TDP-43: NTD – N-terminal domain; NLS – nuclear localization signal; RRM1 (106–177 a.a.) and RRM2 (192–259 a.a.) – RNA-recognition domains; NES – nuclear export signal.

TDP-43 is a protein composed of a N-terminal domain (NTD), two RNA-recognition domains (RRM1 and RRM2) and an amyloidogenic C-terminal Gly-rich domain (Fig. 1). While the latter domain does not have amino acids, which are able to chelate zinc ions, the N-terminal domain and the two RRM domains have 12 and 20 potential zinc chelators (Cys, His and Glu residues) respectively (Fig. 1A). Recently, it has been suggested that conformational changes in RRM2 may play a role in aggregation and toxicity of the protein^28^. Therefore, we hypothesized that RRM domains of TDP-43 are able to bind zinc ions and that this binding might affect protein conformation and its propensity to aggregate. In the present study, using calorimetric and spectral methods we demonstrated that zinc direct binding to TDP-43 fragment 102–269 (RRM12) resulted in RRM12 aggregation into rope-like structures. Furthermore, these structures were positive to Thioflavin-T (ThT) fluorescence, implying that they could correspond to amyloid nuclei. Our findings support the hypothesis of a direct implication of zinc ions in the mechanism of TDP-43 aggregation.

## Results

### RRM12 of TDP-43 binds zinc ions

**Figure 2:**
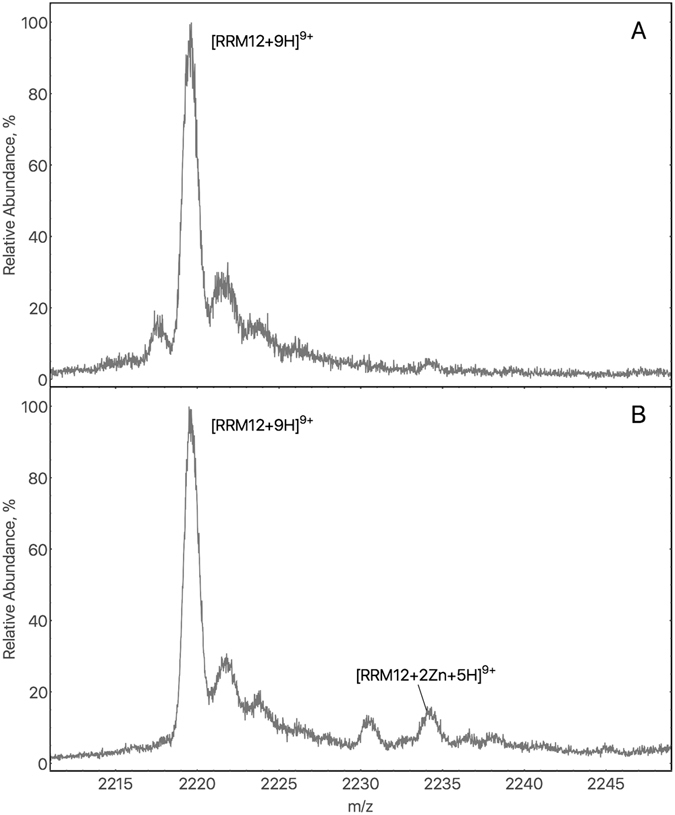
(**A**) Mass Spectrum of 25 µM RRM12 in the absence of zinc ions. Zoom on the 9+ charge state ion (2219.7 m/z) from a six-charge state envelope. Calculated average mass for the major peak is 19967.7 ± 1 Da (the theoretical mass of RRM12 is 19967.5 Da). (**B**) Mass Spectrum of 25 µM RRM12 in the presence of two-fold excess of zinc ions. A new peak at 2234.1 m/z, calculated average mass 20098 ± 1.4 Da, correspond to the species [RRM12 + 2Zn^2+^ + 5 H^+^]^9+^ (theoretical mass 20099.5 Da).

Electrospray ionization mass spectrometry (ESI-MS), which allows to detect non-covalently bound complexes, was used to obtain evidence of zinc interaction with the RRM12 region of TDP-43. MS experiments were carried out in 50 mM Tris-HCl pH 7.3 in the presence of 2-fold excess of zinc ions (50 µM). Comparison of mass spectra of RRM12 in the absence and in the presence of zinc ions, revealed the existence of a zinc-bound species of RRM12 (Fig. 2). A new peak, at 2234.1 m/z, corresponds to the species [RRM12 + 2Zn^2+^ + 5 H^+^]^9+^ with the calculated average mass 20098 ± 1.4 Da (theoretical mass 20099.5 Da). The peak observed at 2230 m/z corresponding to a species with an average calculated mass that is 98 Da higher than the main peak, it is likely to result from non-covalent attachment to protein ions of sulfuric or phosphoric acid coming from trace amounts of sulfate or phosphate salts in the analyte solution^29^. To increase the relative intensity of these species, we increased the zinc concentration, resulting in an overall drop in signal intensity. To determine if zinc interaction impacted RRM12 tertiary structure we investigated its thermostability using differential scanning fluorimetry.

### Zinc decrease thermostability of RRM12

**Figure 3:**
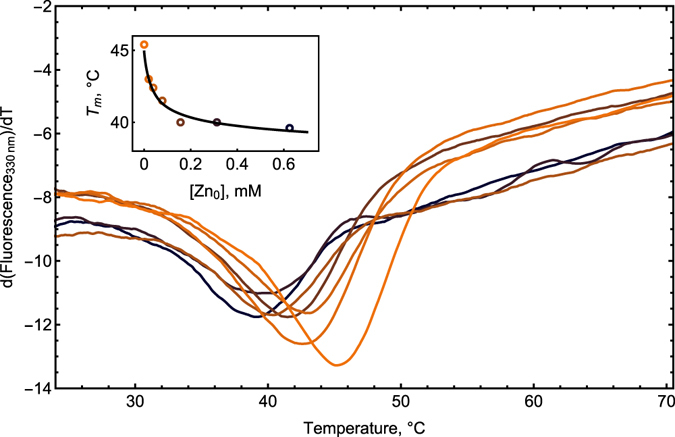
First derivative of thermal unfolding curves of 24 µM RRM12 in 50 mM Tris buffer at pH 7.3 obtained using Prometheus NT.Plex instrument (NanoTemper Technologies GmbH) in the presence of zinc ions at 0; 20; 39; 78; 156; 310; 625 μM (shown in colors from orange to black). Insert: Denaturation temperatures *T*_*m*_ plotted vs. total zinc concentration [*Zn*_*0*_] in circles and best fit as solid black curve.

To study the thermostability of RRM12 we followed the variations in the fluorescence signal upon heating. These variations are correlated with changes in the local environment of aromatic amino acids, allowing us to determine the thermostability of the RRM12 tertiary structure. The first derivative of the temperature dependence of the intrinsic fluorescence signal of RRM12 at 330 nm in the presence of different concentrations of zinc ions is shown in Fig. 3. Apo-form of RRM12 denatures in a single transition peak at the temperature *T*_*m*_ = 45.2 ± 0.6 °C. Taking into account that 330 nm corresponds to tryptophan and tyrosine emission and that these amino acids are mostly located in the RRM1 domain (Fig. 1), we can conclude that the *T*_*m*_ is associated with the thermostability of this domain. This is in good agreement with recently published data for the thermostability of TDP-43 fragment 1–256 a.a. monitored by circular dichroism^30^. Progressive increase of zinc ion concentrations from 20 to 625 μM resulted in the decrease of RRM12 thermostability, as shown by the gradual shift of the denaturation peak to lower temperatures down to 40.0 ± 0.2 °C (Fig. 3). Interestingly while most divalent ions binding to proteins generally increases their thermostability^31, 32^ zinc ions often decrease protein thermostability^33, 34^. As a negative control of unspecific influence of divalent ions on RRM12 thermostability, calcium ions were used. The presence of Ca^2+^ did not impact RRM12 thermostability in a large range of concentrations (data not shown).

To estimate the zinc binding constant, we used experimental values of denaturation temperature *T*_*m*_ at different concentrations of zinc ions. Using the equation linking the shift in melting temperature to the binding constant^35^, we fitted the experimental data (Fig. 3, insert) and obtained a value for the constant of zinc binding to RRM12 of 1.0 ± 0.5 × 10^6^ M^−1^. To further characterize the thermodynamics of this interaction isothermal titration calorimetry (ITC) was used.

### Thermodynamics of RRM12/zinc interaction

**Figure 4:**
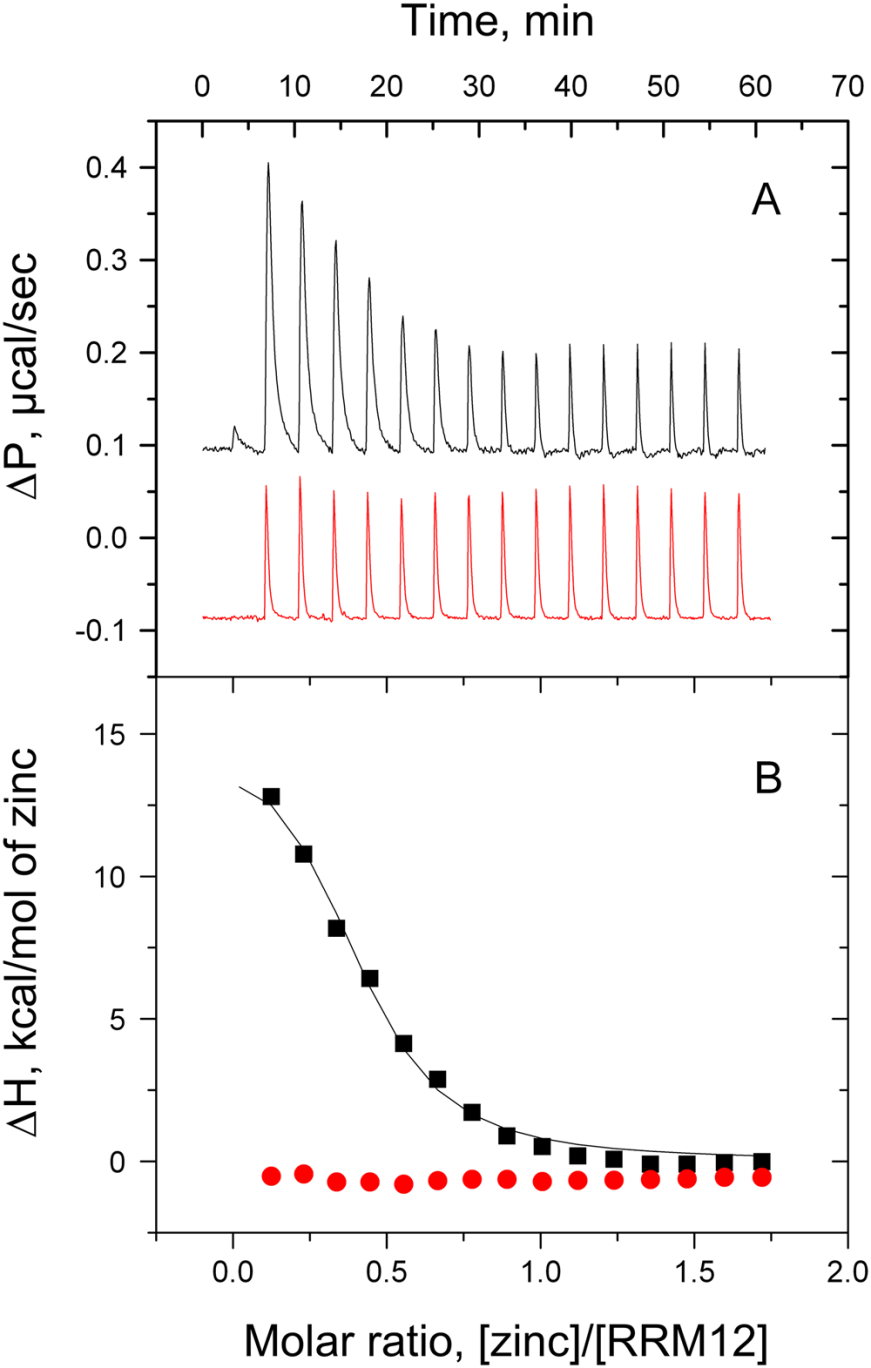
Typical ITC titration curves (**A**) and binding isotherms (**B**) for zinc (black) and calcium (red) interactions with 60 µM of RMM12 at 42 °C in 50 mM Tris, 1 mM TCEP, pH 7.3.

No significant heat exchange was detected at 10, 25 and 37 °C upon RRM12 titration by zinc (data not shown), indicating that enthalpy of binding *ΔH* is close to zero. Thus, to increase the *ΔH* we conducted ITC experiments at 42 °C (Fig. 4, black curve). Indeed at 42 °C, as we showed by DSF, the binding of zinc leads to RRM12 unfolding. The *ΔH* is thus apparent since it comprises the heat of unfolding of RRM12 upon zinc binding. Control titration of RRM12 by calcium ions did not result in any signal that could be associated with interaction (Fig. 4, red curve). Fitting experimental data with a “one set of sites” model allowed to determine all thermodynamic parameters of interaction (*K*_*a*_ = 2.8 ± 0.5 × 10^5^ M^−1^; *∆H* = 15.2 ± 0.8 kcal M^−1^; *∆S* = 73.6 cal M^−1^ K^−1^; *N* = 0.4 ± 0.1). The zinc-binding constant obtained using ITC is close to the constant we estimated earlier from thermostability experiments (1.0 ± 0.5 × 10^6^ M^−1^).

### Zinc induces aggregation of RRM12

**Figure 5:**
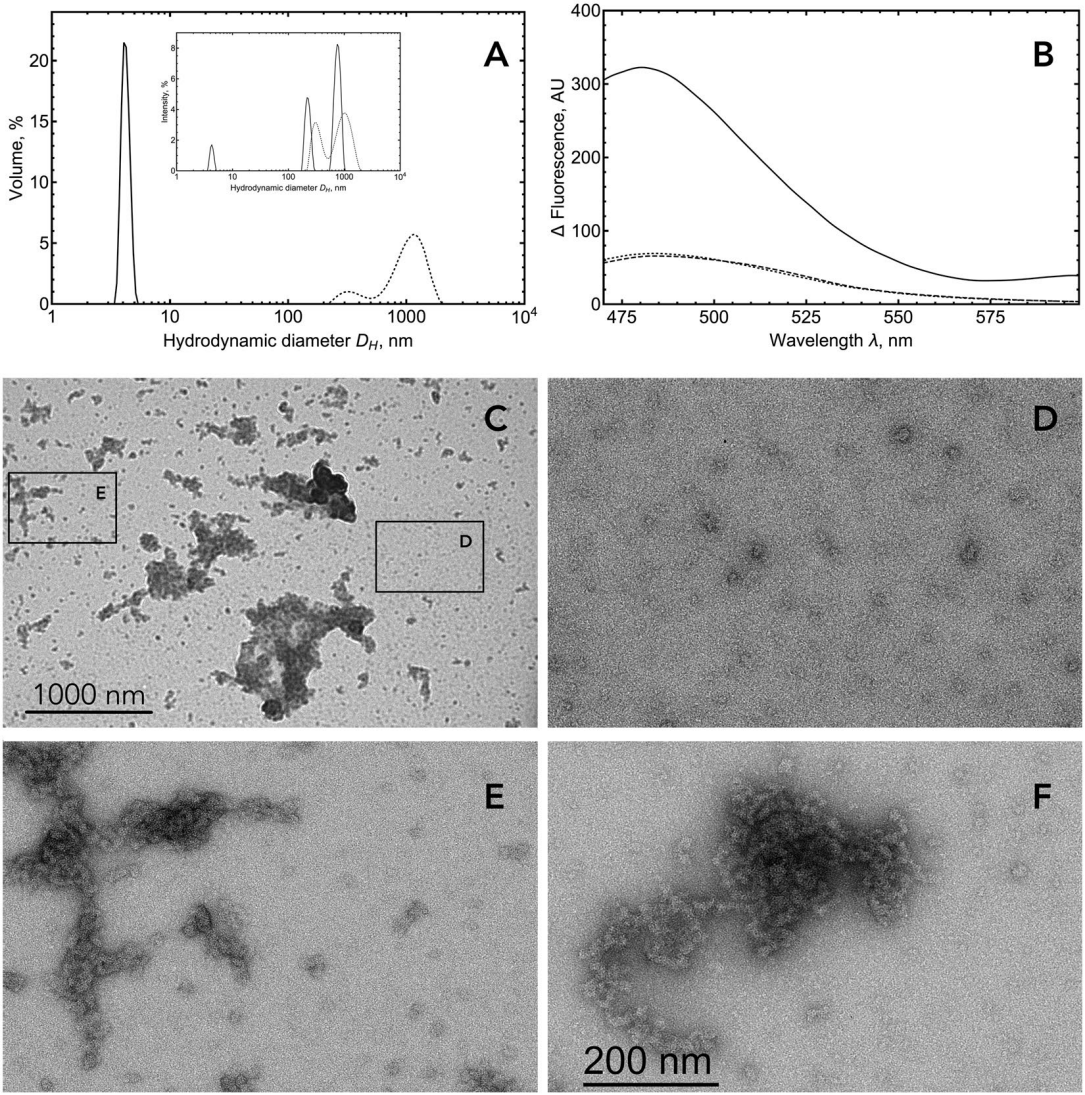
(**A**) DLS analysis of RRM12 using Zetasizer NanoS Malvern instrument. Size distribution by volume and intensity (insert) of 10 µM RRM12 in the absence (solid curve) and in the presence of 10 µM zinc ions (dotted curve). (**B**) Fluorescence spectra of 10 µM RRM12 sample in the absence (dashed line) and in the presence of 10 µM zinc ions (solid line). ThT concentration was 18 µM. Control spectra for ThT alone in buffer (dotted curve). (**C**) Representative electron micrographs of negatively stained TDP-43 aggregates and zoomed-in images of small oligomers (**D**) and tangled rope-like aggregates (**E**,**F**).

**Figure 6:**
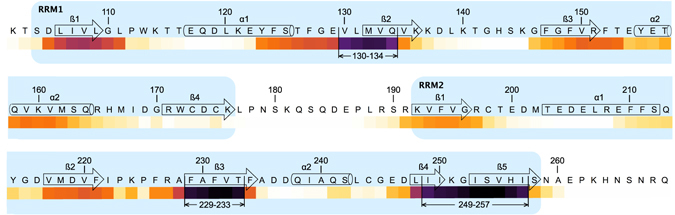
“Hot spots” prediction of amyloidogenic segments within TDP-43 RNA-recognition domains RRM1 and RRM2 (amino-acids 102 to 269). Amyloidogenicity prediction was performed using four independent methods *i*.*e*. Aggrescan^55^, Pasta 2.0^56^, Tango^57–59^ and Salsa^60^. Results obtained from each method were standardized as a percentage considering that the maximum score given by the method was 100%. The average of the standardized percentages was calculated for each amino acid, converted in percentage, and transcribed as a “white-yellow-orange-purple-black” color scale. Amyloidogenic segments 130–134, 229–233, and 249–257 were determined setting a threshold of 80%. Secondary structures were drawn from TDP-43 structure (PDBID: 4BS2)^36^. α-helix and β-strands are schematized by cylinders and arrows, respectively, and are numbered in each RRM domain to make comparison easier with ref. 47. RRM1 and RRM2 are shown in light blue.

To obtain evidence of protein aggregation we monitored this process using dynamic light scattering (DLS). In the absence of zinc ions, RRM12 is mostly represented in solution by a non-spherical monomer species with a hydrodynamic diameter of 5.56 nm, which is in good agreement with its resolved NMR structure (PDBID: 4BS2)^36^. While the intensity distribution of particle sizes (Fig. 5A, insert) shows the presence of some amount of aggregates with hydrodynamic diameters from 200 to 800 nm in the solution, the volume distribution of particle sizes (Fig. 5A) demonstrates that this amount is negligible. In the presence of an equimolar concentration of zinc ions, we observed the complete disappearance of the monomer species and the appearance of several high oligomeric species with hydrodynamic diameters from 300 to 1000 nm (Fig. 5A). Chelation of zinc ions by large excess of EDTA (up to 5 mM), was not able to reverse this process and RRM12 stayed oligomerized (data not shown). To characterize RRM12 oligomers formed in the presence of zinc ions, we used Thioflavin-T (ThT), the most common marker of amyloid fibrils that shows a strong fluorescence increase upon binding to amyloid aggregates. Fluorescence experiments (Fig. 5B) demonstrated that RRM12 oligomers formed in the presence of zinc have amyloid characteristics. Further investigation of formed structures by electron microscopy revealed two different types of oligomers: large aggregates with linear size from 100 to 1000 nm (Fig. 5C), which are in a good agreement with our DLS experiment and much smaller oligomers with linear size from 20 to 30 nm (Fig. 5D). Nevertheless, the total amount of RRM12 in the form of small oligomers was minor since they were not observed using DLS. Their contribution to the size distribution by volume in DLS is negligible. The electron microscopy zoom of RRM12 aggregates revealed the existence of particular shapes that looked like tangled (Fig. 5F) and twisted (Fig. 5E) rope-like structures. To highlight RRM12 segments that could be involved in amyloid-like structure formation, the RRM12 sequence was analyzed by four predictive methods to detect amyloid aggregation “hot spots” (Fig. 6). Obtained results clearly highlighted the three prone-to-aggregate segments within RRM12: _130_VLMVQ_134_, _229_FAFVT_233_ and _249_IIKGISVHI_257_.

## Discussion

There is a whole class of aggregation-prone proteins associated with different proteinopathies, i.e. neurodegenerative disorders with protein aggregation-based pathology^22^. In healthy cells, these proteins are soluble and do not exhibit pathogenic properties. They usually play an important role in different cell processes: SOD1 is implicated in oxidative reactions, FUS and TDP-43 in RNA metabolism, and Tau protein regulates microtubule dynamics by interacting with tubulin^37^. Different endogenous factors such as mutations, hyper phosphorylation, isomerization and other post-translational modifications could favor the transition of these proteins into pathological conformations triggering their aggregation. Remarkably, most of these proteins, namely Tau, amyloid-beta, SOD1, synucleins, and FUS/TLS, bind zinc ions. Moreover, zinc was found to play a key role in pathogenesis of several neurodegenerative diseases (NDs) by inducing protein aggregation. Thus, if not the causative agent, zinc is considered as an important factor that is implicated in the development of pathological protein aggregation in some NDs.

TDP-43 inclusions were found in several neurodegenerative diseases including ALS, FTLD, AD and Parkinson’s disease^38^. Over the past few years the aggregation of TDP-43, its fragments and amyloidogenic peptides have been extensively studied *in vivo* and *in vitro*. Until now, there was only one publication pointing to a possible implication of zinc ions in the pathological aggregation of TDP-43. It was demonstrated that zinc induces depletion and aggregation of endogenous TDP-43 in cells^20^. Here we found that most probably the mechanism of this aggregation implicates direct binding of zinc ions to RNA recognition motives of TDP-43. Our data suggests that zinc ions bind to TDP-43 and impact tertiary structure of RRMs. Thus, as in the case of other previously mentioned aggregation-prone proteins, zinc binding could play an important role both in TDP-43 regulation and aggregation. Nevertheless, further investigations are necessary to find the exact role of zinc in the TDP-43 physiological and pathological context.

The total zinc concentration in human cells are known to be around 10^−4^ M^39^, while attempts to measure free zinc in cells have produced a wide range of concentrations from 10^−5^ to 10^−12^ M depending on the approach^40^. From 30 to 40% of total cell zinc is located in the nucleus^41^ and the local zinc concentration can vary by several order of magnitude. In the central nervous system, zinc is highly present and plays a role of neurosecretory product or cofactor. It is highly concentrated in the synaptic vesicles of some types of neurons^42^. Many zinc metalloproteins have metal binding affinities in the nM to pM range. However, mM affinity zinc binding sites are also abundant in the cell^40^. For example, another zinc-dependent nuclear protein S100A2 has an affinity for zinc ions in the micromolar range^43^, as we found for the TDP-43 RRM12 domain.

Our MS data clearly showed the presence of a RRM12 species bound with two zinc ions, thus we expected that using ITC we would obtain a stoichiometry of zinc binding to RRM12 close to two. However, by ITC we found a stoichiometry less than one. It is thus reasonable to assume that the registered ITC signal corresponds to only one of the two zinc binding sites on RRM12, and that the enthalpy of binding for the second site remained undetectable. The low stoichiometry of this site (0.4 ± 0.1) could be explained by RRM12 concomitant aggregation in the presence of zinc resulting in a decrease of the binding sites accessibility. For this site, the zinc/RRM12 interaction is enthalpy unfavorable (*∆H* > 0) and entropy driven (*∆S* > *0*) at 42 °C. The appearance of a calorimetric signal at 42 °C can be explained by the partial unfolding of RRM12 in the presence of zinc at this temperature. Indeed, zinc binding destabilizes the RRM12 structure and while at 42 °C zinc-free protein is mostly folded, at the same temperature zinc-bound RRM12 is partially unfolded. This unfolding is accompanied by an additional heat exchange that enabled us to follow the zinc binding and determine an association constant of 2.8 ± 0.5 × 10^5^ M^−1^, which is close to the value of 1.0 ± 0.5 × 10^6^ M^−1^ we had estimated in our thermostability experiments.

**Figure 7:**
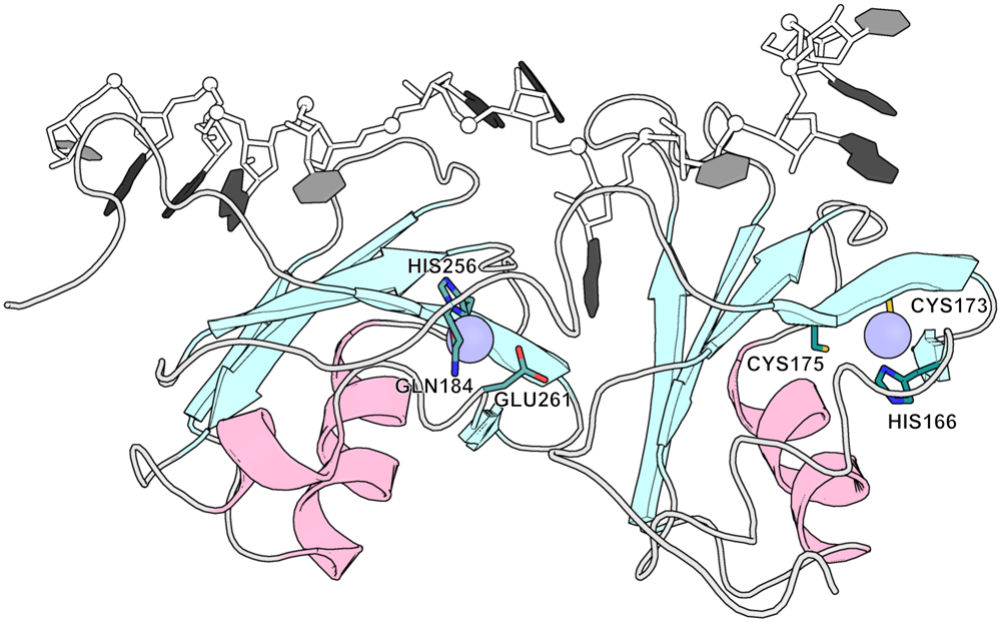
Hypothesized TDP43-zinc complex calculated using existing NMR structure of TDP43-RNA complex (PDBID: 4BS2). RNA is presented with shadows of gray. TDP43 secondary structure elements presented with pale cyan (for β-sheets) and light pink (for α-helixes). Two putative zinc locations are presented with light blue circles and surrounding residues with sticks where oxygen, nitrogen and sulfur atoms colored with red, blue and yellow respectively.

Unfortunately, there are no structural data of zinc binding to TDP-43. Nevertheless, using the published NMR structure of the RRM12 domain in complex with RNA we can highlight some regions as potential zinc-binding sites. By searching in this structure for closely located cysteine and histidine amino acids, we found one potential zinc-binding site formed by Cys175, Cys173 and His166 in the last β-strand of RRM1 and another one in RRM2 domain formed by His256, Glu261 and Cys244 (Fig. 7). This potential site is located on the surface close to a highly flexible C-terminus, which also has several potential zinc chelators, including His264. More in depth research is now necessary to confirm the localization of zinc binding sites on RRM domains of TDP-43 and determine the amino acids implicated in the chelation of zinc.

Taking into consideration that zinc is able to induce the aggregation of endogenous TDP-43 in cells^20^, we could also hypothesize that, as for other aggregation-prone proteins implicated in ND, pathological aggregation of TDP-43 could be also linked to its zinc-binding properties. Indeed, zinc-induced aggregation of RRM12 could be linked to the destabilization of its structure in the presence of zinc ions and partial unfolding of RRM12 at near-physiological temperatures. Since zinc readily forms stable coordination complexes, the majority of intracellular zinc is bound to proteins and free zinc concentrations are found to be low^44^. Still, oxidative stress associated with neurodegenerative diseases^45^ may lead to the release of zinc ions^44, 46^, resulting in local increases in zinc concentration promoting denaturation and aggregation of TDP-43. Finally, we demonstrated that aggregates of RRM12 formed in the presence of zinc ions are ThT-positive and identified three amyloid aggregation amino acid “hot spot” segments within RRM12. One “hot spot” _130_VLMVQ_134_, is located within the TDP-43 RRM1 domain, and two others _229_FAFVT_233_ and _249_IIKGISVHI_257_ within β3 and β5 of RRM2. The peptides, which contains “hot spots” from RRM2 have been shown to form long straight sheet-like fibrils that failed in the induction of ThT fluorescence^47^. In our case, we did not observe any fibril formation, but we found that aggregates were positive to ThT. Thus, we hypothesized that “hot-spots” located within RRM1 could be responsible for amyloid-like aggregation of RRM12. It should be noted that, although initially ALS inclusions were reported as aggregates with lack of amyloid features^48^, recent studies found that some TDP-43 positive inclusions stain positive for amyloid dyes^11, 49, 50^ which is in line with our findings.

In summary, we demonstrated that the RRM12 domain of TDP-43 binds zinc ions with an association constant of 2.8 ± 0.2 × 10^5^ M^−1^. This binding significantly decreases thermostability of the RRM12 domain and induces the formation of amyloid-like aggregates, pointing to potential implication of zinc ions in physiological functions and pathological propensities of TDP-43.

## Materials and Methods

### Protein purification

Plasmid for purification of RNA-binding domain of TDP-43 (residues 102–269) was generously provided by Peter J Lukavsky. Protein was purified as described previously^36^. It was expressed from a pET-28a vector introduced into BL21(DE3) grown at 37 °C in LB medium “CONDA”, with 50 µg/mL of kanamycin. When absorbance at 600 nm reached 0.6, protein overexpression was induced by the addition of 1 mM IPTG. After 5 h induction at 20 °C, cells were pelleted and resuspended in 20 mM Hepes, 1 M NaCl, 10% glycerol, 20 mM Imidazole, 5 mM β-mercaptoethanol, 8 mM Triton, pH 7.5 with anti-proteases. After 2 runs in French Press (6 tones), lysate was cleared by centrifugation at 43000 g for 30 min at 4 °C. Supernatant was filtered through a 0.45 µm filter and loaded onto a 5 mL Hitrap crude FF (GE Healthcare) equilibrated in buffer A (20 mM Hepes, 1 M NaCl, 10% glycerol, 20 mM Imidazole, 5 mM β-mercaptoethanol, pH 7.5). Histidine tagged TDP43 was eluted with a gradient of buffer B (Buffer A with 300 mM Imidazole) 100% in 30 minutes at 1 mL/min. Histidine tag was removed by the addition of 1 mg of TEV (Sigma Aldrich T5544) in 100 mL of collected protein. The solution was dialyzed overnight at 4      °C against buffer A in a 3.5 kDa MCO membrane and then reloaded on the HiTrap crude FF equilibrated in buffer A. TDP43 without tag was collected in the flow-through and purity was assessed on 12% SDS PAGE Electrophoresis. Before use, protein was dialyzed overnight at 4 °C against metal-free 50 mM Tris, 1 mM TCEP buffer, pH 7.3. Integrity of the protein was checked by mass spectrometry (MS). Average mass measured by MS 19967.7 ± 1 Da fits very well with the theoretical mass of the protein without histidine tag: 19 967,5 Da. No peak was detected at the theoretical mass corresponding to nickel-protein association.

### Mass Spectrometry

Electrospray Q-ToF mass spectrometer (Synapt G1, Waters) was used for native mass spectrometry experiments. Soft ionization parameters were used to prevent in source dissociation of the zinc-protein complexes: spray voltage 3.2 kV, sampling cone 150 and vacuum source increased to 4 mBar. Protein solutions at 25 μΜ with or without 50 µM ZnCl_2_ in 50 mM Tris-HCl pH 7.3, were desalted against ammonium acetate 200 mM using 5 kDa cut-off Spin X (Corning).

### Isothermal titration calorimetry

Thermodynamic parameters of zinc binding to RRM12 were measured using a MicroCal iTC200 System (Malvern, UK) as described previously^51^. Experiments were carried out at 10, 25, 37 and 42 °C in 50 mM Tris, 1 mM TCEP at pH 7.3. Aliquots of 0.5 mM ZnCl_2_ solution (2–2.5 μL) were injected into the 0.2 mL cell containing a solution of 60 µM RRM12 to achieve a complete binding isotherm. Heat of the dilution was measured by injecting the ligand (ZnCl_2_) into the buffer solution; the values obtained were subtracted from the heat of the reaction to obtain the effective heat of binding. Data were analyzed using the MicroCal Origin software and were fitted with a “one set of sites” model that led to the determination of affinity constants (*K*_*a*_), stoichiometry (zinc to protein ratio, *N*) and enthalpy of binding (*ΔH*). Consequently, the entropy variations (*ΔS*) were calculated according to the standard equations. All experiments were repeated at least three times.

### Differential Scanning Fluorimetry

The protein thermostability was measured in the presence of different concentrations of divalent ions in 50 mM Tris, 1 mM TCEP at pH 7.3 using a label-free fluorimetric analysis with a Prometheus NT.Plex instrument (NanoTemper Technologies). NanoDSF grade capillaries were filled with a 25 µM solution of RRM12. Concentration of zinc or calcium ions varied from 19.5 to 625 µM. Capillaries were loaded into the Prometheus NT.Plex and heated from 25 °C to 70 °C with a 1 K/min heating rate at low detector sensitivity with an excitation power of 10%. Unfolding transition points (*T*_*m*_) were determined from the first derivative of the changes in the emission wavelengths of tryptophan fluorescence at 330 nm, which were automatically identified by the Prometheus NT.Plex control software^52^.

### Dynamic Light Scattering

Dynamic light scattering (DLS) experiments were carried out using a Zetasizer Nano S (Malvern Instruments) at 25 °C. Three measurements were performed; each one consisting in 10–15 runs of 10 seconds. The scattering angle was 173°. RRM12 was diluted to 10 µM and centrifuged at 14000 rpm for 20 minutes. RRM12 size was determined in the absence or presence of 20 µM zinc ions. For the determination of the hydrodynamic diameter (D_H_), the provided software uses the Stokes-Einstein relation to obtain the intensity averaged size distribution, which requires the viscosity and refractive index values of the dispersion medium. In our case, we used a viscosity of 0.8878 cP and a refractive index of 1.332 (at 25 °C). We displayed our results as a volume, number and intensity distribution of particle size.

### Fluorescence Thioflavin-T (ThT) assay

RRM12 samples at 60 µM were directly diluted into fluorescence cells containing 250 µL of 20 mM Tris buffer, 20 µM ZnCl_2_, pH 7.5 supplemented with 5 µL of 1 mM ThT. For measurements, RRM12 final concentration was 10 µM. Fluorescence spectra were immediately acquired after the dilution step in 0.2 (excitation direction) ×1 cm (emission) cells (Hellma) thermostated at 25 ± 0.5 °C. Excitation was at 440 nm and emission spectra were recorded from 470 to 600 nm with slit widths of 15/15 nm using a Perkin-Elmer LS 55 fluorescence spectrometer operating at a PM of 800 V.

### Transmission electron microscopy

Four microliters of RRM12 samples at 10 µM were placed on carbon-coated copper grids (300 mesh) during 1 min. After having been blotted, grids were washed with distilled water, blotted again, negatively stained for 30 seconds with 2% (wt/vol) uranyl acetate. The grids were then dried and observed with a JEOL 2200FS transmission electron microscope (Tokyo, Japan) operating at 200 kV. Images were recorded using a 4 k × 4 k slow-scan CCD camera (Gatan, Inc. Pleasanton, USA).

### TDP43 structure analysis

Coordinates for RNA-TDP43 complex NMR derived structure (PDBID: 4BS2)^36^ was obtained from the PDB data bank. The python PMX module was used to parse the atom coordinates^53^. For atoms ND1, NE2 and SG in HIS and CYS residues neighboring atoms with names ND1, NE2, SG, OE1 and OD1 were searched with distances less than 6 Å. False positive contacts for ASN and GLN were filtered and results were ranged on base of neighbors count and printed. Top two were chosen for further discussion. All operations were done with Python program language.
